# Racial and Ethnic Disparities in Tuberculosis Incidence, Arkansas, USA, 2010–2021

**DOI:** 10.3201/eid3001.230778

**Published:** 2024-01

**Authors:** Maheen Humayun, Leonard Mukasa, Wen Ye, Joseph H. Bates, Zhenhua Yang

**Affiliations:** University of Michigan, Ann Arbor, Michigan, USA (M. Humayun, W. Ye, Z. Yang);; Arkansas Department of Health, Little Rock, Arkansas, USA (L. Mukasa, J.H. Bates);; University of Arkansas for Medical Sciences, Little Rock, Arkansas, USA (L. Mukasa, J.H. Bates)

**Keywords:** TB, tuberculosis and other mycobacteria, bacteria, respiratory infections, epidemiology, disparity, race, ethnicity, Arkansas, United States

## Abstract

We conducted an epidemiologic assessment of disease distribution by race/ethnicity to identify subpopulation-specific drivers of tuberculosis (TB). We used detailed racial/ethnic categorizations for the 932 TB cases diagnosed in Arkansas, USA, during 2010–2021. After adjusting for age and sex, racial/ethnic disparities persisted; the Native Hawaiian/Pacific Islander (NHPI) group had the highest risk for TB (risk ratio 173.6, 95% CI 140.6–214.2) compared with the non-Hispanic White group, followed by Asian, Hispanic, and non-Hispanic Black. Notable racial/ethnic disparities existed across all age groups; NHPI persons 0–14 years of age were at a particularly increased risk for TB (risk ratio 888, 95% CI 403–1,962). The risks for sputum smear–positive pulmonary TB and extrapulmonary TB were both significantly higher for racial/ethnic minority groups. Our findings suggest that TB control in Arkansas can benefit from a targeted focus on subpopulations at increased risk for TB.

Globally, tuberculosis (TB) is the 13th leading cause of death and a leading infectious killer, second to COVID-19 (*1*,*2*). In 2021, TB affected 10.6 million persons and caused 1.6 million deaths worldwide (*2*). Moreover, a quarter of the world’s population is latently infected with *Mycobacterium tuberculosis*, which puts them at a 5%–10% lifetime risk of developing active disease (*2*–*4*). Global trends indicate that the World Health Organization’s (WHO) End TB Strategy milestones will likely be missed because TB incidence has reduced by only 10% and TB deaths by 5.9% during 2015–2021, compared with the desired goals of 20% reduction in incidence and 35% reduction in deaths (*2*,*5*). The COVID-19 pandemic has further disrupted TB notification and treatment, reversing the progress toward global TB elimination while widening existing inequalities (*2*,*6*).

The TB epidemic is considered a major challenge in low-resource, high-burden countries; 30 high-burden countries represent 87% of the global burden (*2*). However, the epidemiology of TB in low-incidence countries is characterized by concentration of the TB burden, sometimes as high as in high-burden countries, among socially and historically marginalized populations (*7*,*8*). Before the impact of COVID-19 on TB notification, TB incidence continued to decline during 1993–2019 in the United States; however, the annual rate at which TB declined plateaued during the later years (*9*). Similarly, a recent study from Arkansas reported no significant decline in TB incidence during 2009–2020 (*10*). To move toward preelimination (1 case/100,000 population) and eventually elimination (<1 case/100,000 population), current TB interventions should be adapted to unique local challenges focusing on populations at increased risk for TB as suggested by WHO’s action framework for TB elimination in low-incidence countries (*7*,*8*). This framework calls for epidemiologic assessment of disease distribution in the local population by important sociodemographic variables; however, such disaggregated analysis is typically not available in TB surveillance reports, limiting the ability of public health programs to develop pro-equity policies (*7*,*8*,*11*,*12*). 

In the United States, race is a strong social determinant of health because it serves as a proxy for systemic and structural barriers to equitable opportunities for education, employment, earning, housing, and healthcare, which perpetuates racial discrimination and unjust distribution of resources that lead to adverse health outcomes (*13*,*14*). In this study from Arkansas, USA, a state with TB incidence below the national average of 2.4 cases/100,000 population, we quantify the racial/ethnic disparities in TB incidence at the population level using detailed racial/ethnic categorizations that have not been widely used in previous TB studies in Arkansas (*15*–*17*). This study will not only help map subpopulations at an increased risk for TB in a low-burden setting but also guide the development of targeted TB interventions in light of the underlying factors that differentially drive TB incidence across racial/ethnic groups. 

This study used de-identified patient data that we retrospectively retrieved from the TB surveillance database maintained by the Arkansas Health Department. The University of Michigan Health Sciences and Behavioral Sciences Institutional Review Board (IRB-HSBS) determined that this study was not regulated.

## Methods

### Study Population

This study included all 932 TB cases diagnosed in Arkansas during 2010–2021. All of these cases met the Centers for Disease Control and Prevention (CDC)’s definition of a verified TB case. These verified cases met either laboratory or clinical case definition, including those verified by provider diagnosis as described in CDC’s TB case reporting manual (*18*).

### Data Collection and Data Sources

Demographic and clinical data available in this dataset were collected on verified TB cases using the standard CDC TB reporting form. We obtained US Census Bureau official population estimates for 2010–2019 from annual resident population estimates for 6 race groups (5 race-alone groups and 2 or more races) by age, sex and Hispanic origin for states and the District of Columbia from April 1, 2010–July 1, 2019; April 1, 2020; and July 1, 2020. We obtained population estimates for 2020 and 2021 from annual state resident population estimates for 6 race groups (5 race-alone groups and 2 or more races) by age, sex, and Hispanic origin from April 1, 2020–July 1, 2021 (*19*).

### Data Analysis

This study had 4 objectives. The objectives were to characterize racial/ethnic disparities in TB risk; to determine if the observed racial/ethnic disparities were a result of underlying differences in sex and age distribution; to track age-specific incidence for racial/ethnic groups to draw inferences related to the underlying drivers of TB incidence; and to characterize racial/ethnic differences in advanced TB disease at diagnosis.

From the TB surveillance dataset, we created a combined variable for race and ethnicity with 5 categories so that we did not consider racial/ethnic identities in isolation (*20*). The Hispanic category included all racial subcategories. We categorized non-Hispanic persons into Asian, non-Hispanic Black, Native Hawaiian/Pacific Islander (NHPI), and non-Hispanic White categories. We did not include American Indian/Alaska Native (n = 4) and multirace (n = 1) categories in race/ethnicity-stratified results because of their small sample sizes. We categorized age as 0–14 years, 15–24 years, 25–44 years, 45–64 years, and >65 years. Consistent with the definition used by CDC, US-born persons included those who were eligible for US citizenship at the time of birth regardless of place of birth.

To address the first objective, we calculated the overall TB incidence for the period 2010–2021, with corresponding 95% CI for the state and the 5 racial/ethnic groups mentioned. We calculated TB incidence per 100,000 population using the population estimates from the US Census Bureau (*19*). We estimated TB incidence in the overall population and stratified by race/ethnicity and age group using Poisson regression with an offset term for the total population size. To further characterize TB-related disparities, we calculated risk ratio (RR) estimates for the race/ethnicity-combined variable, sex, and age group using Poisson regression. Because age and sex are important determinants of TB risk, we calculated race/ethnicity RRs that were adjusted for both age and sex concurrently using Poisson regression to achieve our second objective. In the absence of genotyping data, age-specific TB incidence can help draw inferences related to the underlying mechanisms that drive TB incidence. Previous studies demonstrated that TB among older adults strongly reflects reactivation of latent TB infection (LTBI) in low-burden settings, whereas TB is typically a result of recent transmission among young children (*21*,*22*). Hence, the third objective determined how racial disparities tracked across age groups by reporting RRs for race/ethnicity from age-specific Poisson models adjusted for sex. In addition, to track age disparities within each racial/ethnic group, we reported RRs for age groups from race/ethnicity-specific Poisson models while adjusting for sex.

Pulmonary TB (PTB) often starts with minimum infiltrate and progresses to additional infiltrate; sputum smear positivity has been used previously as a proxy for delayed diagnosis (*17*). The occurrence of extrapulmonary TB (EPTB) often reflects the spread of *M. tuberculosis* outside of the lungs due to the host’s inability to contain the infection (*23*). We evaluated sputum smear–positive PTB and EPTB as important indicators for advanced disease. For those 2 outcomes, we calculated RRs and adjusted RRs for the race/ethnicity-combined variable, sex, and age group using Poisson regression.

We used the non-Hispanic White category as the reference group for race/ethnicity and female as the reference category for sex because those groups were at the lowest risk for TB compared with other variable categories. For age, no one category was at the lowest risk for TB across stratified results, so we used the >65 years age category as the reference group to follow how TB risk progressed with age. We determined model fit using a goodness-of-fit test. We conducted statistical analysis using the SAS OnDemand for Academics (SAS Institute Inc., https://www.sas.com).

## Results

### Characteristics of Study Sample

Among the 932 TB cases in our study, 72% were bacteriologically confirmed through either nucleic acid amplification test or positive culture (Table 1). Most TB cases (76.5%) were exclusively diagnosed as PTB patients. Among all TB patients, 35.73% had a positive sputum smear result and 43.2% of the patients with PTB diagnosis (including those with both PTB and EPTB) had a positive sputum smear result. Of the total study sample, 86.6% of TB patients identified as non-Hispanic with diverse racial categorizations; 63.2% of the study patients were male. Most case-patients among Asian (91.7%), NHPI (67.4%), and Hispanic (82.4%) persons were not US born, and all of the non–US-born NHPI TB case-patients in this study were born in the Marshall Islands. For the non-Hispanic White group, only 2.3% of TB cases were non–US born, and for the non-Hispanic Black group, only 6.1% of TB cases were non–US born.

**Table 1:** Demographic and clinical characteristics of 932 TB patients diagnosed in Arkansas, USA, during 2010–2021*

Characteristic	No. (%)
Diagnosis confirmation	
** Bacteriologically confirmed**	669 (71.78)
** Clinically diagnosed**	263 (28.22)
TB disease site	
** Pulmonary**	713 (76.50)
** Extrapulmonary**	164 (17.60)
** Both**	55 (5.90)
Sputum smear result	
** Positive**	333 (35.73)
** Negative**	473 (50.75)
** Not done**	126 (13.52)
Race/ethnicity	
** Hispanic**	125 (13.41)
** Asian**	109 (11.70)
** Non-Hispanic Black/African American**	245 (26.29)
** Native Hawaiian/Pacific Islander**	138 (14.81)
** Non-Hispanic White**	310 (33.26)
** American Indian or Alaska Native**	4 (0.43)
** Multirace**	1 (0.11)
Sex	
** F**	336 (36.05)
** M**	589 (63.20)
** Unknown**	7 (0.75)
Age group	
** 0–14 y**	92 (9.87)
** 15–24 y**	85 (9.12)
** 25–44 y**	253 (27.15)
** 45–64 y**	282 (30.26)
** ≥**65 y	220 (23.61)
Origin of birth	
** US born**	613 (65.77)
** Non-US born**	319 (34.23)
Year	
** 2010**	76 (8.15)
** 2011**	85 (9.12)
** 2012**	71 (7.62)
** 2013**	72 (7.73)
** 2014**	93 (9.98)
** 2015**	90 (9.66)
** 2016**	91 (9.76)
** 2017**	85 (9.12)
** 2018**	76 (8.15)
** 2019**	65 (6.97)
** 2020**	59 (6.33)
** 2021**	69 (7.40)

*Percentages may not total 100 because of rounding. TB, tuberculosis.

### Racial/Ethnic Disparity in TB Incidence

The overall TB incidence in Arkansas was 2.6 (95% CI 2.4–2.8) cases/100,000 population during 2010–2021 (Table 2). Upon stratifying by race/ethnicity, the NHPI persons (131.6 [95% CI 111.4–155.5] cases/100,000 population) had the highest incidence of TB followed by Asian (20.0 [95% CI 16.6–24.2] cases/100,000 population), Hispanic (4.8 [95% CI 4.0– 5.7] cases/100,000 population), non-Hispanic Black (4.4 [95% CI 3.9–5.0] cases/100,000 population), and non-Hispanic White persons (1.2 [95% CI 1.0–1.3] cases/100,000 population).

**Table 2:** Average TB incidence by race/ethnicity, Arkansas, USA, 2010–2021*

Category	Incidence (95% CI)					
	All ages	0–14 y	15–24 y	25–44 y	45–64 y	>65 y
State	2.58 (2.42–2.76)	1.29 (1.05–1.59)	1.75 (1.42–2.17)	2.76 (2.44–3.13)	3.09 (2.75–3.48)	3.79 (3.32–4.33)
Hispanic	4.75 (3.99–5.67)	1.40 (0.80–2.47)	3.64 (2.26–5.86)	6.27 (4.76–8.28)	7.51 (5.25–10.75)	16.61 (10.02–27.56)
Asian	20.02 (16.60–24.16)	1.79 (0.45–7.14)	18.52 (11.17–30.72)	25.45 (19.29–33.59)	29.85 (21.33–41.77)	19.45 (9.73–38.90)
Non-Hispanic Black	4.42 (3.90–5.01)	1.65 (1.07–2.53)	2.98 (2.04–4.35)	4.45 (3.50–5.66)	6.34 (5.11–7.88)	8.54 (6.44–11.33)
NHPI	131.62 (111.39–155.51)	139.60 (105.51–184. 71)	108.08 (68.94–169.44)	109.27 (80.45–148.40)	206.17 (139.31–305.11)	158.67 (59.55–422.75)
Non-Hispanic White	1.17 (1.04–1.31)	0.16 (0.07–0.33)	0.22 (0.10–0.46)	0.66 (0.49–0.89)	1.50 (1.24–1.81)	2.88 (2.44–3.39)

*Incidence is no. cases/100,000 population. The table provides overall and age-stratified TB incidence for the state of Arkansas and for racial/ethnic groups as calculated by Poisson regression. NHPI, Native Hawaiian/Pacific Islander; TB, tuberculosis.

### Racial/Ethnic Disparity after Adjusting for Sex and Age Differences

Based on the unadjusted model (Table 3), the risk for TB was many folds higher for all racial/ethnic groups when compared with the non-Hispanic White group. The risk for TB for NHPI persons was 113 (95% CI 92.1–137.7) times the risk for non-Hispanic White persons. Asian (RR 17.1, 95% CI 13.8–21.3) Hispanic (RR 4.0, 95% CI 3.3–5.0), and non-Hispanic Black (RR 3.8, 95% CI 3.2–4.5) persons all had higher risk for TB than non-Hispanic White persons. Male persons were at an 81% (RR 1.8, 95% CI 1.6–2.1) higher risk for TB than female persons. The risk for TB was 66% (RR 0.3, 95% CI 0.3–0.4) lower for the youngest group, 0–14 years of age, compared to the oldest age group. TB risk increased with age; the >65-year age group had the highest risk for TB.

**Table 3:** Disparity in tuberculosis incidence by race, sex, and age, Arkansas, USA, 2010–2021*

Covariate	**Unadjusted risk ratio (95%CI)**	**Age- and sex-adjusted risk ratio (95%CI)**
Race/ethnicity†		
Hispanic	4.07 (3.30–5.01)	5.94 (4.79–7.37)
Asian	17.14 (13.77–21.33)	21.61 (17.32–26.98)
Non-Hispanic Black	3.78 (3.20–4.48)	4.60 (3.88–5.45)
NHPI	112.64 (92.13–137.73)	173.58 (140.64–214.24)
Non-Hispanic White	Referent	Referent
Sex‡		
F	Referent	Referent
M	1.81 (1.59–2.07)	1.88 (1.64–2.15)
Age group§		
0–14 y	0.34 (0.27–0.44)	0.16 (0.12–0.20)
15–24 y	0.46 (0.36–0.59)	0.23 (0.18–0.30)
25–44 y	0.73 (0.61–0.87)	0.36 (0.30–0.43)
45–64 y	0.82 (0.68–0.97)	0.58 (0.49–0.70)
>65 y	Referent	Referent

*RR calculated by Poisson regression. NHPI, Native Hawaiian/Pacific Islander; RR, risk ratio. †Using unadjusted RR model 1. ‡Using unadjusted RR model 2. §Using unadjusted RR model 3.

After adjusting for age group and sex, racial/ethnic disparities continued to persist. NHPI persons were at the highest risk for TB compared with non-Hispanic White persons (RR 173.6, 95% CI 140.6– 214.2), followed by Asian (RR 21.6, 95% CI 17.3– 27.0), Hispanic (RR 5.9, 95% CI 4.8– 7.4), and non-Hispanic Black (RR 4.6, 95% CI 3.9–5.5) persons.

### Age-Related Racial/Ethnic Disparities in TB Incidence

The statewide TB incidence was highest among the >65-year age group (3.8 [95% CI 3.3– 4.3] cases/100,000 population) whereas the youngest group, 0–14 years, had the lowest incidence (1.3 [95% CI 1.1–1.6] cases/100,000 population). Risk for TB increased with age in Arkansas (Table 2). The 0–14-year age group had the lowest risk for TB compared with the ≥65 year age group for non-Hispanic White (RR 0.05, 95% CI 0.02– 0.11), non-Hispanic Black (RR 0.18, 95% CI 0.11–0.30), Asian (RR 0.09, 95% CI 0.02–0.42), and Hispanic (RR 0.08, 95% CI 0.04–0.18) groups, (Table 4). We observed no significant age differences for the NHPI group (p = 0.13). NHPI persons 0–14 years of age had 888 (95% CI 403–1,962) times and NHPI persons >65 years of age had 55 (95% CI 20–148) times the risk of TB compared to similarly aged non-Hispanic White persons (Table 5). 

**Table 4:** Age disparities in tuberculosis incidence across racial/ethnic groups, Arkansas, USA, 2010–2021*

Age group	Risk ratio (95% CI)				
	Non-Hispanic White, no. cases = 305	Non-Hispanic Black, no. cases = 244	Asian, no. cases = 109	NHPI, no. cases = 138	Hispanic, no. cases = 124
0–14 y	0.05 (0.02–0.11)	0.18 (0.11–0.30)	0.09 (0.02–0.42)	0.87 (0.31–2.42)	0.08 (0.04–0.18)
15–24 y	0.07 (0.03–0.15)	0.33 (0.20–0.52)	0.92 (0.39–2.16)	0.68 (0.23–1.99)	0.21 (0.11–0.43)
25–44 y	0.22 (0.15–0.31)	0.50 (0.34–0.72)	1.27 (0.60–2.68)	0.68 (0.24–1.90)	0.37 (0.21–0.65)
45–64 y	0.50 (0.39–0.64)	0.72 (0.50–1.03)	1.51 (0.70–3.26)	1.29 (0.45–3.72)	0.44 (0.24–0.82)
>65 y	Referent	Referent	Referent	Referent	Referent

*Risk ratio calculated by Poisson regression. NHPI, Native Hawaiian/Pacific Islander.

**Table 5:** Racial/ethnic disparities in tuberculosis incidence across age groups, Arkansas, USA, 2010–2021*

Race/ethnicity	Risk ratio (95% CI)				
	0–14 y, no. cases = 91	15–24 y, no. cases = 85	25–44 y, no. cases = 249	45–64 y, no. cases = 277	≥65 y, no. cases = 218
Hispanic	8.93 (3.52–22.68)	16.68 (6.92–40.22)	9.37 (6.22–14.13)	4.84 (3.23–7.26)	5.57 (3.27–9.48)
Asian	11.38 (2.36–54.78)	85.02 (34.67–208.53)	38.81 (25.75–58.50)	20.72 (14.08–30.50)	7.09 (3.48–14.46)
Non-Hispanic Black	10.50 (4.46–24.69)	13.77 (6.00–31.63)	6.83 (4.64–10.06)	4.36 (3.26–5.81)	3.06 (2.21–4.25)
NHPI	888.70 (402.55–1961.98)	496.40 (208.68–1180.84)	162.91 (105.93–250.55)	138.01 (89.26–213.38)	54.82 (20.30–148.08)
Non-Hispanic White	Referent	Referent	Referent	Referent	Referent

*RR calculated by Poisson regression. The table reports RR estimates with 95% CI while adjusting for sex. NH, non-Hispanic; NHPI, Native Hawaiian/Pacific Islander; RR, risk ratio.

### Racial/Ethnic Disparity in Advanced Disease at Diagnosis

The risk for sputum smear-positive PTB was highest for NHPI persons (RR 138.8, 95% CI 94.7–203.7), followed by Asian (RR 14.4, 95% CI 9.5–22.0), Hispanic (RR 5.5, 95% CI 3.8–8.0), and non-Hispanic Black (RR 4.8, 95% CI 3.7–6.3) persons when compared with non-Hispanic White persons (Table 6). The risk for EPTB was highest for NHPI persons (RR 133.3, 95% CI 83.7–212.4), followed by Asian (RR 31.4, 95% CI 21.1–46.7), Hispanic (RR 5.3, 95% CI 3.3–8.3), and non-Hispanic Black (RR 4.5, 95% CI 3.1–6.3) persons compared to non-Hispanic White persons (Table 6).

**Table 6:** Disparities in advanced TB disease at diagnosis by race/ethnicity, sex, and age, Arkansas, USA, 2010–2021*

Covariate	RR (95% CI) for sputum smear-positive PTB			RR (95% CI) for EPTB	
	Unadjusted	Age- and sex-adjusted		Unadjusted	Age- and sex-adjusted
Race/ethnicity†					
** Hispanic**	3.42 (2.39–4.90)	5.54 (3.84–8.00)		3.61 (2.31–5.66)	5.26 (3.32–8.34)
** Asian**	11.07 (7.28–16.82)	14.43 (9.46–22.02)		25.97 (17.59–38.35)	31.37 (21.09–46.66)
** Non-Hispanic Black**	3.80 (2.90–4.99)	4.80 (3.65–6.31)		3.68 (2.59–5.22)	4.46 (3.13–6.34)
** NHPI**	78.73 (54.40–113.94)	138.84 (94.65–203.67)		89.90 (57.41–140.78)	133.32 (83.68–212.41)
** Non-Hispanic White**	Referent	Referent		Referent	Referent
Sex‡					
** M**	2.27 (1.79–2.87)	2.40 (1.89–3.05)		1.43 (1.09–1.87)	1.49 (1.14–1.95)
** F**	Referent	Referent		Referent	Referent
Age group§					
** 0–14 y**	NA**¶**	NA		0.20 (0.11–0.36)	0.10 (0.05–0.18)
** 15–24 y**	0.42 (0.27–0.64)	0.22 (0.14–0.34)		0.37 (0.22–0.64)	0.20 (0.11–0.35)
** 25–44 y**	0.73 (0.54–0.99)	0.37 (0.27–0.51)		0.93 (0.66–1.31)	0.48 (0.33–0.69)
** 45–64 y**	1.04 (0.78–1.38)	0.75 (0.56–1.00)		0.66 (0.45–0.96)	0.48 (0.33–0.71)
>65 y	Referent	Referent		Referent	Referent

*Estimates used Poisson regression, EPTB includes cases concurrently diagnosed with PTB. EPTB, extrapulmonary tuberculosis; NA, not available; NHPI, Native Hawaiian/Pacific Islander; PTB, pulmonary tuberculosis; RR, risk ratio; TB, tuberculosis. †Using unadjusted RR model 1. ‡Using unadjusted RR model 2. §Using unadjusted RR model 3. ¶Not available due to small sample size.

## Discussion

The TB incidence in the United States and Arkansas has been <10 cases/100,000 population for several years; however, neither the state nor the country has been able to make the final push toward preelimination and elimination targets. The underlying epidemiologic factors that drive the remaining TB epidemic in low-incidence countries differ from those in high-burden settings and also across subpopulations within low-incidence settings, which suggests the need for locally informed, context-specific TB interventions (*24*).

Our study provides an in-depth epidemiologic understanding of the concentrated TB epidemic in Arkansas that is not well captured by aggregated statewide estimates. We found remarkable disparities in TB incidence around the axes of race/ethnicity, sex, and age. Of particular importance were racial/ethnic disparities, which could not be explained by age and sex differences across racial/ethnic groups. Age-specific TB incidence and differences in clinical manifestation of TB at diagnosis across racial/ethnic categories hold important lessons for understanding the drivers of TB incidence and challenges related to health equity in Arkansas.

The racial/ethnic disparities that we observed in our study are consistent with previous studies conducted in Arkansas and the United States, which consistently reported racial disparities in risk for LTBI, recent transmission, and TB disease (*13*,*15*,*16*,*25*,*26*). In 2021, a total of 88.1% of the TB cases reported in the United States were attributable to racial/ethnic minorities (*9*,*27*). Such observed racial disparities can be explained as a consequence of structural racism that perpetuates health inequities primarily through 2 interlinked pathways: residential segregation and inadequate healthcare (*14*,*28*). Persons from racial/ethnic minority groups are more likely to live in neighborhoods with high population density, limited healthcare access, poor housing conditions, and greater air pollution, which makes them more susceptible to acquiring TB infection (*14*,*28*,*29*). Moreover, those persons are more likely to experience conditions such as diabetes, HIV, and malnutrition that can contribute toward progression to TB disease (*28*,*30*). In essence, race is associated with socioeconomic status (SES), including generational wealth, income, and education, which then mediates the relationship between race and susceptibility to infection and progression to disease through psychosocial stress, nutrition, physical environment, healthcare access, and immune function (*28*,*31*).

The NHPI persons in Arkansas had a strikingly high TB incidence of 131.6 cases/100,000 population, many times higher than their annual national-level TB incidence rate that has remained <20 cases/100,000 during 2003–2021 (*9*,*32*). Most of the TB case-patients among NHPI in our study were born in the Marshall Islands, where TB incidence was 280.6 cases/100,000 population in 2020 and 343.2 cases/100,000 population in 2021 (*9*,*32*). Decades of colonial rule and testing of nuclear weapons by the US government during 1946–1958 had socioeconomic repercussions for the health infrastructure of the Marshall Islands, where the prevalence of comorbidities that substantially increase TB risk is alarmingly high (*33*). Under the Compact of Free Association, the people of the Marshall Islands can freely travel, live, and work in the United States, where they experience language, cultural, and economic barriers when accessing healthcare that can lead to infection, delayed diagnosis, and prolonged infectiousness with implications for community transmission (*34*,*35*). Despite the high incidence of TB in the Marshall Islands, persons from that country are not required to undergo screening for LTBI or active disease upon arrival to the United States (*36*). Screening those persons for TB may help in early diagnosis and treatment, thereby reducing the burden of TB in Arkansas and among NHPI persons.

TB is driven by various mechanisms, mainly by reactivation of LTBI or primary disease by a recent transmission, each of which requires specialized mitigation strategies (*37*). TB incidence was 139.6 cases/100,000 population among 0–14-year-old NHPI children in our study; incidence was<2 cases/100,000 population among all other racial/ethnic groups of similar age. The risk among 0–14-year-old NHPI children was 888 times the risk among non-Hispanic White children of similar age. Because progression from infection to disease is rapid, TB among young children is a good marker for ongoing community transmission (*38*,*39*). Given the high risk for sputum smear positive PTB and elevated TB risk among 0–14-year-olds, TB transmission appears to play a role in driving TB incidence among NHPI persons. Hence, to curtail the disproportionate TB burden for NHPI persons, mitigation strategies should focus on active case finding in addition to LTBI screening of adults to disrupt chains of transmission (*36*). Curtailing the TB epidemic will also require ramping up contact tracing based on contact disclosure from TB patients, who often hesitate to name contacts because of stigma around TB in their communities (*40*). Community-level advocacy and awareness using culturally appropriate tools can improve contact disclosure and equip the local community with the necessary information on TB diagnosis, treatment, and transmission (*40*).

For Hispanic, non-Hispanic White, and non-Hispanic Black persons, the risk for TB was highest among the oldest age group, indicating that TB is likely driven by reactivation of LTBI in those subpopulations (*41*). Two previous studies conducted in Arkansas using genotyping data from 1997–2010 demonstrated that TB incidence among >65-year-olds was largely driven by nonclustered TB incidence, which is indicative of reactivation of LTBI (*15*,*16*). In addition, foreign-born non-Hispanic White, non-Hispanic Black, Asian, and Hispanic persons had significantly higher risks for TB than did US-born persons (Appendix Table). The high risk among foreign-born persons likely indicates reactivation, as suggested by a previous national-level study based on genotyping data (*42*). Racial/ethnic groups with a high proportion of foreign-born TB cases can particularly benefit from TB control efforts focused on preventing reactivation of LTBI.

Sputum smear–positive PTB is highly infectious because its high bacillary load leads to an elevated risk for transmission (*43*,*44*). In addition, the increased risk of smear-positive PTB at first diagnosis in persons from racial/ethnic minority groups likely points toward differential access to timely and adequate TB care, previously supported by a study from Arkansas, and such difference can result in severe disease with poor health outcomes (*17*,*44*). Another study from Arkansas found that TB patients from rural Arkansas faced delays in receiving the correct diagnosis and were treated for other conditions for several months (*40*). Our findings suggest the need to explore the barriers related to TB care that affect various subpopulations in Arkansas. Increasing awareness of TB among healthcare workers, especially in a time when TB incidence is low in the country can help equip them with the knowledge needed to make timely and accurate TB diagnosis (*40*).

The increased risk for EPTB, including concurrent PTB, among persons from racial/ethnic minority groups is indicative of elevated risk for advanced disease that is a diagnostic and a therapeutic challenge due to the dissemination of the disease (*45*). An increased prevalence of comorbidities such as HIV might predispose racial/ethnic minority groups to EPTB, suggesting the need for improved management of risk factors that compromise host immunity (*46*). The Arkansas Office of Minority Health and Health Disparities reported that among non-Hispanic Black persons, the mortality rate for HIV was 6 times higher and for diabetes was 2 times higher than the rates among non-Hispanic White persons during 2011–2015 (*47*). TB preventive strategies should go beyond curtailing transmission and focus on improved comanagement of noncommunicable conditions, which are often modifiable risk factors, by collaborating across health programs to provide more holistic patient-centered care (*48*).

This study relies on surveillance data that provides access to limited study variables. We used markers of advanced TB disease to make inferences related to access to timely and adequate care in Arkansas. To clarify the factors that limit access to timely care, future studies should collect qualitative data crucial for determining delays in healthcare and assessing if these delays are patient- or provider-related to bridge health inequities related to TB care. Distinguishing between ongoing community transmission and reactivation of remotely acquired TB infection is crucial when designing TB interventions, but lack of genotyping data in this study prevented reliable evaluation of the relative contribution of each of these 2 mechanisms across racial/ethnic groups (*49*). Despite those limitations, this study provides incidence and RR estimates stratified by detailed racial/ethnic categories that had not been previously reported at the population level for Arkansas using the most recent surveillance data (*11*,*15*–*17*). 

The state-level estimates of TB incidence in Arkansas are misleading because the progress toward TB elimination is unequally felt across racial/ethnic subpopulations. Our findings demonstrate that drivers of TB incidence vary across subpopulations, which necessitates designing context-specific TB interventions. Although our results may not be generalizable to other low-incidence settings, the racial/ethnic disparities we observed demonstrate the need for detailed disaggregated analysis of TB surveillance data by race/ethnicity while providing a framework for such an analysis in other US states.

Appendix Additional information about racial and ethnic disparities in tuberculosis incidence, Arkansas, United States, 2010–2021.
