# Supplementary material for: Racial and Ethnic Disparities in Tuberculosis Incidence, Arkansas, USA, 2010–2021
Source: Emerg Infect Dis. 2024 Jan;30(1):116–24. doi: 10.3201/eid3001.230778 (PMC10756389; doi:10.3201/eid3001.230778)
Supplement: Appendix — Additional information about racial and ethnic disparities in tuberculosis incidence, Arkansas, United States, 2010–2021. [file 23-0778-Techapp-s1.pdf]

Article DOI: http://doi.org/10.3201/eid3001.230778

EID cannot ensure accessibility for supplementary materials supplied by authors. Readers who have difficulty accessing supplementary content should contact the authors for assistance.

Racial and Ethnic Disparities in Tuberculosis Incidence, Arkansas, USA, 2010–2021

Appendix

To obtain U.S. born and foreign-born population estimates, the American Community Survey (ACS) 1-year Public Use Microdata Sample (PUMS) files were used for all years except 2020. Due to the COVID-19 impact, 1-year ACS Public Use Microdata Sample (PUMS) data file was not available for 2020 and was replaced with 1-year ACS Public Use Microdata Sample (PUMS) with experimental weights. For 2020, ACS 1-year PUMS with Experimental Weights was used. Poisson regression was used to calculate nativity RRs for NH White, NH Black, Asian and Hispanic groups. For NHPI group, negative binomial regression was used to calculate nativity RR as Poisson regression model did not provide a good fit.

Appendix Table. Nativity risk ratio with 95% CI across racial/ethnic groups, Arkansas, United States, 2010–2021*

Nativity	NH White	NH Black	Asian	NHPI	Hispanic
U.S. born	Ref	Ref	Ref	Ref	Ref
Non–U.S. born	2.93 (1.38–6.20)	8.89 (5.27–14.98)	5.11 (2.58–10.10)	1.69 (0.85–3.36)	7.19 (4.54–11.40)

*NH, non-Hispanic; NHPI, Native Hawaiian/Pacific Islander; Ref, reference.
